# Impaired myocardial deformation and ventricular vascular coupling in obese adolescents with dysglycemia

**DOI:** 10.1186/s12933-019-0976-0

**Published:** 2019-12-19

**Authors:** Preneet Cheema Brar, Anne Chun, Xiazhou Fan, Vivek Jani, Mary Craft, Puneet Bhatla, Shelby Kutty

**Affiliations:** 1 Division of Endocrinology and Diabetes, Department of Pediatrics, NYU Grossman School of Medicine, New York, NY 10016 USA; 2 Division of Cardiology, Department of Pediatrics, NYU Grossman School of Medicine, New York, NY 10016 USA; 3 NYU Grossman School of Medicine, New York, NY 10016 USA; 4 Division of Pediatric Cardiology, University of Nebraska College of Medicine, Omaha, NE 68918 USA; 5 Helen B. Taussig Heart Center, The Johns Hopkins Hospital and School of Medicine, 1800 Orleans Street, Baltimore, MD 21205-2196 USA

**Keywords:** Obesity, Abnormal glucose tolerance, Longitudinal and circumferential strain and strain rate, Ventricular vascular coupling, Two-dimensional speckle-tracking echocardiography, Echocardiography

## Abstract

**Background:**

It is unknown that dysglycemia in obese adolescents has effects on myocardial deformation that are more pronounced when compared to obesity alone. We hypothesized that obesity associated abnormal glucose tolerance (dysglycemia) would have adverse effects on two-dimensional speckle tracking echocardiography derived longitudinal, radial and circumferential strain (LS, RS, CS) compared to age and gender lean controls. We also examined if changes in deformation would be reflected in abnormal ventricular vascular coupling indices (VVI).

**Methods:**

In a prospective cross-sectional design 39 obese adolescents (15.9 ± 1.7 years; 101.5 ± 39 kg; female − 58%) were compared to age and gender matched lean controls (15.7 ± 1.8 yrs, 60 ± 12.8 kg). Based on results from an oral glucose tolerance test (OGTT), obese adolescents were categorized as obese normoglycemic (ONG, n = 25) or obese dysglycemic (ODG, n = 14). Left ventricular (LV) global and average LS, CS, RS and strain rate were measured. LV ejection fraction and mass index were measured and VVI approximated as ratio of arterial elasticity (Ea) and end-systolic elastance (Ees).

**Results:**

Adolescents with ODG had significantly (P = 0.005) impaired global LS (− 20.98% ± 2.8%) compared to controls (− 23.01% ± 2.3%). A similar (P = 0.0027) reduction was observed in average LS for adolescents with ODG (18.87% ± 2.5%) compared to controls (20.49% ± 2%). Global CS was also decreased (P = 0.03) in ODG (− 23.95%) compared to ONG (− 25.80). A similar trend was observed in average CS after multivariate regression for BMI and blood pressure. CS correlated with HbA1c in both groups (P = 0.05). VVI had a negative correlation with both LS (r = − 0.4, P = 0.025) and CS rate (r = − 0.36, P = 0.04).

**Conclusions:**

Myocardial strain and strain rate were significantly altered in obese adolescents. Unfavorable subclinical reductions in global and average CS were more pronounced in adolescents with dysglycemia compared to obese adolescents with normoglycemia and controls. These data indicate progressive worsening of subendocardial function across the spectrum of glucose tolerance. Strain rate was predictive of VVI in obese adolescents, suggesting strain rate may be a sensitive marker for cardiac remodeling in abnormal glucose homeostasis states.

## Background

The pediatric obesity epidemic had resulted in an exponential rise of Type-2 diabetes mellitus (T2DM). It is well established that in children and adolescents with obesity, ventricular chamber size, left ventricular (LV) wall thickness, and LV mass are elevated compared with lean pears despite no significant changes in ejection fraction (EF) [1]. Furthermore, myocardial geometry and function as assessed by a two-dimensional and three-dimensional speckle tracking echocardiography (2D-STE, 3D-STE) is impaired in obesity with significant reductions in LV circumferential strain, longitudinal strain, and strain rate [1–4]. In obese states, high body mass index (BMI), insulin resistance and hyperinsulinemia are the most important predictors of subclinical myocardial deformational changes [5].

In states of abnormal glucose tolerance (prediabetes) and/or T2DM, myocardial deformational changes are worsened due to the hyperglycemia, increased fatty acid acids, activation of the renin angiotensin system, microangiopathy and increased oxidative stress. In the context of obese adolescents with abnormal glucose tolerance (dysglycemia), LV mass, systolic blood pressure, and resting heart rate are observed to be elevated when compared to lean peers. The study of obese adolescents who have glucose dysregulation (dysglycemia) merits investigation of subclinical myocardial changes in the context of pediatric obesity [6, 7]. However, it is unknown whether dysglycemia in comorbid adolescent obesity has effects on myocardial deformation parameters that are more pronounced when compared to obesity alone.

2D-STE and 3D-STE have recently emerged as reliable techniques for quantification of myocardial deformation in multiple imaging planes due to its angle independence and high interobserver reliability [8, 9]. High temporal resolution during image acquisition allows for the determination of strain rate, or the rate of myocardial deformation, which correlates with LV peak elastance, a load independent measure of LV function, and diastolic ventricular filling [8–10]. In pediatric populations, control values for myocardial longitudinal strain and strain rate from 2D-STE are well established [10]. LV global longitudinal strain (GLS), the most validated of myocardial deformation indices, is a measure of subendocardial longitudinal myofiber function and is susceptible to ischemia and fibrosis [11]. Changes in LV GLS are well documented in ischemic cardiomyopathy, although not relied upon clinically [12]. In addition to its association with ischemia, decreased LV GLS has been reported early in the disease process in adolescents with T1DM and adults with T2DM [13, 14]. Furthermore, the degree of LV GLS impairment correlates well with HbA1c levels, suggesting that glycemic control may be on the main risk factors for impairment of myocardial mechanics [13, 14]. While exercise induced LVEF depression has been observed in a subpopulation of young adults with DM, clinical diabetic cardiomyopathy is not observed in these subjects due to lack of inotropic and microvascular abnormalities; rather, these changes are likely due to impaired ventricular-vascular coupling [15]. Together, these data suggest that LV myocardial deformation parameters and ventricular vascular coupling ratio may serve to be useful subclinical indices of myocardial dysfunction in the context of dysglycemia.

To our knowledge, no studies have investigated myocardial strain and strain rate in adolescents with prediabetes, and few studies have demonstrated changes in LV GLS in T2DM [13]. The objective of this observational cross-sectional study is therefore to compare myocardial mechanics from 2D-STE, namely longitudinal strain, circumferential strain, strain rate, and ventricular-vascular coupling index in obese-normoglycemic and obese-dysglycemic adolescents. We hypothesized the following: (1) Obese adolescents with dysglycemia would have impaired strain parameters (LS and CS) when compared to obese adolescents with normoglycemia and lean controls; (2) In obese adolescents, changes in strain (LS and CS) and strain rate (LSR and CSR) would reflect changes in ventricular vascular coupling.

## Methods

### Study design

A total of 39 obese adolescents with BMI ≥ 95th percentile were recruited for this observational cross-sectional study, as previously reported [16]. All subjects were adolescents recruited from Endocrine and Primary care clinics affiliated within the New York University Langone Medical Center and Health and Hospital Corporation Hospitals (Bellevue and Woodhull) seen for obesity related concerns, namely prediabetes and/or type 2 diabetes, dyslipidemia or hypertension. Written informed consent and age-appropriate assent were obtained from all the subjects before participation and the study was approved by the New York University Institutional Review Board. Recruitment began in October 2011 and ended in February 2014. All study measurements were conducted at the Clinical and Translation Research Center (CTSI) of New York University Langone Medical Center. Participants were instructed to come after a 12 h overnight fast to the Clinical Research Center at the NYU Clinical and Translational Science Institute. A total of n = 6 participants was previously diagnosed with T2DM. All remaining participants not diagnosed with T2DM (n = 33) underwent an oral glucose tolerance test (OGTT) using 75 g of glucose. Samples for glucose, insulin, and cytokines were stored at − 80 °C at the Research Center of New York University.

Key inclusion criteria were: (1) age 12–18 years and (2) Tanner stage II or above. Key exclusion criteria were: (1) Use of medications known to affect insulin sensitivity such as high-dose inhaled glucocorticoids, thiazolidinediones and atypical antipsychotics, (2) medications for treatment of dyslipidemia such as statin therapy, bile acids and fibrates, (3) pregnancy, (4) significant psychiatric illness, (5) blood pressure over 90% for height (6) renal insufficiency based on Schwartz equation as > 0.55 for females and males > 0.7 ages 13–18 years and (7) nonalcoholic fatty liver (NAFLD: AST and ALT ≥ threefold higher value above normal at baseline.

Adolescents were grouped by their OGTT results as obese normoglycemia (ONG; n = 25) and obese dysglycemia (ODG; n = 14). Abnormal glucose tolerance was defined as either (1) impaired fasting glucose (fasting glucose level ≥ 100 mg/dl) or (2) impaired glucose tolerance (2-h postprandial glucose ≥ 140 mg/dl after glucose challenge). None of the adolescents who underwent the OGTT were diagnosed with T2DM after the OGTT.

Adolescents diagnosed with T2DM did not undergo an OGTT and were categorized as ODG. The adolescents with T2DM were treated with metformin and/or insulin. Those on metformin were asked to stop metformin 72 h prior to their study day as metformin can affect vascular studies (flow mediated dilatation), which were performed during the study visit, previously reported [16].

Healthy age and sex-matched controls were derived from the Pediatric Cardiology program at the collaborating center, as part of a registry collecting normative data of strain parameters. The control group consisted of children and adolescents who responded to an institutional IRB approved advertisement for participation. In the control group, 2D-STE was performed for research purposes. The control inclusion criteria included: (1) age 1 to 18 years; (2) no history of heart disease, hypertension, or any other systemic disease. Age appropriate assents were signed by the adolescents with parental consent. Data collected at the time of 2D-STE included gender, date of birth, height, weight, heart rate, and systemic blood pressure. BSA was calculated using the Haycock formula. All participants in the control group had no history of congenital heart disease and were lean BMI < 85% with no signs of glucose intolerance. Males were observed to be heavier in the control cohort (M: F; mean ± SD − 60.5 ± 2.1 and 59.5 ± 8.6 kg respectively). Glycemic status was not established in the control using a glucose tolerance test though there was no history of polyuria or polydipsia elicited by the adolescents.

### Echocardiography

Transthoracic echocardiography images were obtained by an experienced sonographer on a Philips iE33 ultrasound imaging system with a 5 MHz probe (Philips Healthcare, Andover, Massachusetts). Standard echocardiography acquisition included two-dimensional, color flow Doppler, pulsed Doppler, and continuous wave Doppler. 3–5 cardiac cycles were acquired for each measurement with the patient in supine and left lateral decubitus positions per routine clinical protocol. Two dimensional apical 4 chamber and 2 chamber views, as well as parasternal short axis views, were acquired for strain and strain rate analysis. Left ventricular mass was calculated via Devereux’s formula and by 5/6 area-length methods [17]. LV mass index (LWMI) was calculated by dividing LV mass by height^2.7^. Parameters of diastolic function were measured, including mitral valve inflow E & A wave peak velocities, tissue Doppler imaging (TDI) of the mitral valve medial and lateral annulus e′ and a′ wave peak velocities, pulsed Doppler measurements of the right upper pulmonary vein S, D, and A wave peak velocities, and the left atrial volume by biplane Simpson’s method [45, 46]. MV E/A ratio, and lateral and medial mitral E/e′ were also calculated. LAVI > 25 ml/ht^2.7^ was considered to be dilated. MV E/e′ ratio > 8 was considered to be abnormal diastolic function. Due to the limited dataset, diastolic dysfunction was defined by an abnormality in any of these parameters. Studies were de-identified and stored on a Siemens SyngoDynamics DICOM system (Siemens Healthcare, Erlangen, Germany).

### Ventricular-vascular coupling index

Ventricular vascular coupling index (VVI) was estimated in a manner similar to that presented by Sanz et al. [18]. Briefly, VVI is the ratio between LV end-systolic elastance (Ees) and effective arterial elasticity (Ea). LV end systolic elastance was approximated as,

1 $$E_{es} = \frac{ESP}{{ESV - V_{0} }},$$

where ESP is the LV end systolic pressure, ESV is the end systolic volume, and V_0_ is the theoretical volume of the unloaded ventricle, determined as the intercept o the linear ESP-ESV relation. End systolic pressure was approximated from systolic blood pressure as, ESP = 0.9 × SBP^18^. Effective arterial elasticity was approximated as,

2 $$E_{a} = \frac{ESP}{SV},$$

where ESP is end systolic pressure and SV is stroke volume. VVI is therefore determined as VVI = Ea/Ees.

### Deformation analysis

Analysis to derive left ventricular longitudinal, circumferential and radial deformation and strain by offline post processing (Image-Arena Version 4.6 Build 4.6.2.12, Unterschleissheim, Tomtec, Germany) was performed on two-dimensional DICOM image datasets, described elsewhere [19, 20]. Briefly, strain is the fractional change in the length of myocardial segment, and strain rate is determined as the rate of change in strain (s^−1^). S and SR were obtained using standard 2D multiform B-mode grayscale LV images acquired in the apical four, two, and three (3CV) chamber views. S and SR were also obtained in the parasternal short-axis views of the LV at three levels, namely the basal, mid, and apical LV, corresponding to LV short axis sections at the mitral valve (MV), papillary muscles (PM) and LV apex (AP), respectively. A frame rate > 60 Hz was used for all analyses. Five consecutive cardiac cycles at the same heart rate triggered to the R wave of ECG complex were digitally stored in a cine-loop format for offline analysis. Five values were obtained for each strain index (S and SR) and averaged. The timing of aortic valve closure and mitral valve opening with respect to peak S and SR was ascertained from manual inspection of a single PW or CW Doppler from the LV outflow tract. All LV images in the apical and short axis views were obtained at the same heart rate. A region-of-interest width and when necessary was automatically acquired, which was manually adjusted to include the entire myocardial wall. The tracking algorithm followed speckles in the myocardium throughout the cardiac cycle. Results from the tracking algorithm were confirmed by visual inspection, and the number of LV myocardial segments was maximized. By convention, the myocardial strain is obtained from three planes in the LV, namely longitudinal, circumferential, and radial. The corresponding strain indices that were determined include longitudinal strain (LS), longitudinal strain rate (LSR), circumferential strain (CS), circumferential strain rate (CSR), radial strain (RS), and radial strain rate (RSR). All strains were reported as dimensionless percentages (%). LS was calculated as the % change in LV length, which was determined for three individual apical long axis views, namely the 4CV, 3CV, and 2CV. Global longitudinal strain (GLS) was calculated as the average of LS and LSR from all considered views. CS and RS were calculated as the % change in LV circumference and wall thickness, respectively. Both CS and RS were assessed from the LV short axis view at three levels, namely basal (MV), mid ventricular (PM), and distal LV at the apex (AP). Regional CS and RS were obtained from six segments in three parasternal short axis views of the LV, which were averaged to obtain global CS and RS. The software automatically divided the LV short axis views into six segments, namely the anteroseptal, anterior, lateral, posterior, inferior, and sepal segments, as per standard guidelines [23]. SR, the time derivative of S expressed in s^−1^ was obtained at the peak temporal spread of the strain rate acquisition at systole. The peak systolic SR was obtained for each cardiac segment from the same views as strain, discussed in detail above.

### Statistical analysis

**Fig. 1:**
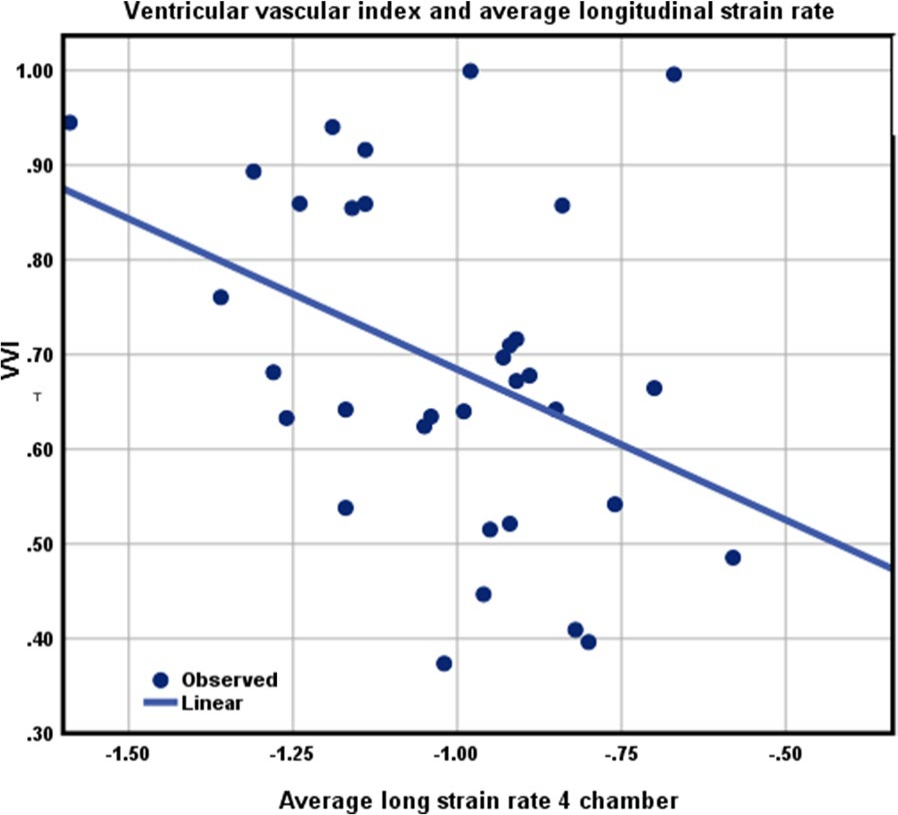
Relationship between VVI and average longitudinal strain rate

**Fig. 2:**
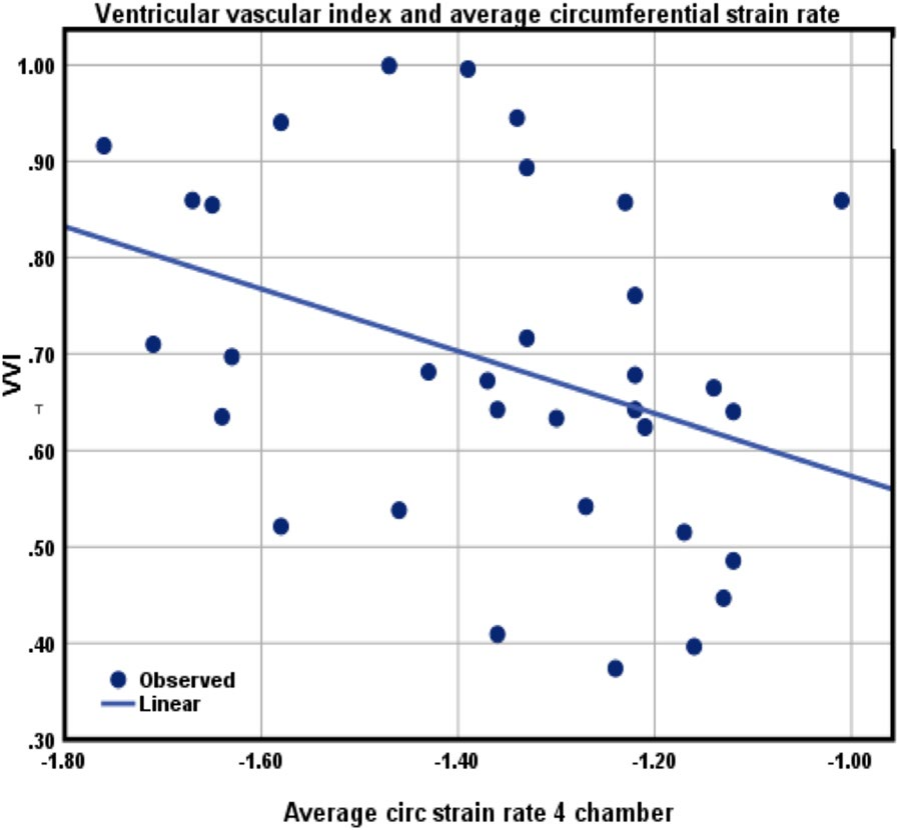
Relationship between VVI and average circumferential strain rate

Data were summarized as frequency for categorical variables, mean ± standard deviation (SD) for normally distributed continuous variables, and median and IQR for continuous data with a skewed distribution. Differences between groups were assessed using the unpaired *t* test or the Wilcoxon rank-sum test for normally and non-normally distributed continuous variables, respectively. Differences between categorical variables were assessed using Fisher’s exact test. Logistic regression was used to assess the univariate associations between presence of strain parameters and study variables. Linear regression was used to illustrate the correlation between average LSR/CSR and VVI (Fig. 1, 2). Multivariable associations between myocardial deformation parameters, namely strain and strain rate, and other variables were investigated in a forward-selection model building procedure, while controlling for BMI, DBP, and SBP. All statistical tests were two-sided, with P < 0.05 considered significant. A commercially available statistical software package (SAS v9.3, SAS Institute Inc., Cary, NC) was used for all analyses.

## Results

### Population characteristics

Table 1 summarizes the characteristics of enrolled obese adolescents classified as obese normoglycemia (ONG) and obese dysglycemia (ODG) based on glucose tolerance results from OGTT. Weight and blood pressure were recorded for control subjects, though glucose status was not available. 60% of the cohort was Hispanic. Eight adolescents were found to have prediabetes (IFG and/or IGT) after OGTT. Six adolescents had confirmed T2DM at baseline; thus, a total of 14 adolescents had dysglycemia. There were more males in the ODG group (total = 9; 5 with prediabetes and 2 with T2DM) compared to females (total = 5; 3 with prediabetes and 2 with T2DM). Males were heavier when compared to females (107 kg versus. 93 kg) though they were younger by 6 months when compared to females. T-test P values in Tables 1, 2, 3 reflect differences between ONG and ODG group as control group were matched for age and gender. ODG and ONG groups were matched for BMI (34 ± 7 vs. 37.5 ± 8.8); however, those with dysglycemia were found to have a higher BMI, though not significantly. HbA1c was found to significantly higher (P < 0.001) in the ODG group (7.4 ± 2.3) compared to the ONG group (5.6 ± 0.3), likely because six adolescents were previously diagnosed with T2DM in the ODG group. Significant differences between both ODG and ONG groups were also observed in systolic blood pressure (P = 0.0025).

**Table 1:** Baseline and anthroprometric characteristics of Obese adolescents and lean controls

	ONG (n = 25)		ODG (n = 14)		Controls (n = 39)		*P value
	Mean	SD	Mean	SD	Mean	SD	
Ethnicity							
Hispanic/Latin origin	64%		57%		29%		–
Black/African American	20%		35%		11%		–
Other (White/Asian/other race)	16%		7%		60%		–
Age (years)	16	1.9	15.9	1.6	15.8	1.9	–
Sex							–
Male	28%		64%		41%		–
Female	72%		36%		59%		0.06
Weight (kg)	93	23	110.1	33.4	59.9	12.8	0.06
BMI	34	7	37.5	8.8	19.2	4	
Systolic* (mm/Hg)	113	6.6	121.6	9.3	107	5.5	0.003
Diastolic (mm/Hg)	64	8.6	61	9.3	59	7	0.41
GL0 (mg/dl)	80	8.7	111.8	49.9	NA		0.003
Hba1c	5.6	0.3	7.4	2.3	NA		0.001

*ONG* obese normoglycemia group, *ODG* obese dysglycemia group, *GL0* fasting glucose, * P values are t-test results between the ONG and ODG groups, *BMI* systolic and diastolic BP were adjusted for in multivariate logistic regression (Table 4)

**Table 2:** Echocardiographic data of obese adolescents

	ONG (n = 25)		ODG (n = 14)		P value
	Mean	SD	Mean	SD	
LVM (5/6 AL)	50	10.4	14	11.27	NS
LVMI (m mode)	66	16	71	21	NS
MV E/A	2.2	0.7	1.8	0.5	0.04
LAVI	22	5	25	9	NS
Lateral E/e′	6.2	1.75	6.4	1.3	NS
Medial E/e′	9.2	2.4	8.6	1.3	NS

*LVM (5/6AL)* LV mass indexed to BSA using 5/6 area length measurement, *LVMI (m mode)* LV mass indexed to BSA, *MV E/A* E/A wave ratio, a diastolic index, *LAVI* left atrial volume indexed, *lateral E/e*′ ratio which is mitral valve inflow E wave to tissue Doppler inflow e′ of lateral mitral annulus and medial, *E/e*′ ratio which was ratio of mitral valve E wave to tissue Doppler inflow of medial mitral valve annulus

**Table 3:** Ventricular vascular coupling data of obese adolescents

	ONG (n = 25)		ODG (n = 14)		P value
	Mean	SD	Mean	SD	
ESP (mmHg)	102	5.9	108	8.1	0.022
Ees (mmHg/ml)	3.3	1	3.1	1.2	NS
Ea (mmHg/ml)	2.1	0.7	2.1	0.5	NS
VVI (Ea/Ees)	0.67	0.2	0.7	0.2	NS

*ESP* end systolic pressure (0.9× systolic blood pressure), *Ees* end systolic elastance, *Ea* arterial elasticity, *VVI* ventricular vascular coupling index

### Echocardiographic results

The Mitral E/A ratio was significantly different (P = 0.0025) between ONG and ODG groups, while no significant differences were observed in index LA volume. E/e′ ratio of lateral and medial mitral valve annulus were not significantly different between the two groups. While only male in ONG had evidence of LA dilatation, five adolescents in ODG had signs of LA dilatation-two (mild); one (moderate) and one adolescents (severe). These results are summarized in Table 2. ESP was observed to be elevated in the ODG group (108 ± 8.1) compared to the ONG group (102 ± 5.9), though not significantly (P = 0.22). No significant differences were observed in LVMI and VVI between both groups.

### Strain, strain rate, and VVI

As previously discussed, ESP was different between both ONG and ODG groups (Table 3). Mean VVI was determined to be 0.7 ± 0.2 for all obese adolescents included in the study, indicating no impairment in ventricular-vascular coupling. VVI was however observed to be negatively correlated with average LS (r = − 0.4; P = 0.025) and circumferential strain rate (CSR) (r = − 0.36; P = 0.04, Figs. 1, 2). The negative correlation between VVI and CSR was also observed in ODG adolescents (r = − 0.7; P = 0.01).

### Strain in obese adolescents and age matched lean controls

Table 4 compares 2D-STE derived global and average longitudinal strain (LS), circumferential strain (CS), and radial strain (RS) in obese adolescents and lean age matched controls. Briefly, global LS was observed to be significant reduced (P = 0.0005) by approximately 15% in obese adolescents (− 20.98%) compared to lean controls (− 23%). A similar trend was observed in average LS, which was observed to be significantly reduced (P = 0.0027) by approximately 20% in obese adolescents (− 18.8%) compared to lean control (− 20.5%). Results remained significant on multivariate logistic regression analysis for both global LS (P = 0.0045) and average LS (P = 0.014). However, no significant differences were observed in global and average CS and RS between both groups. Global LS were higher in males than females (− 20.1 versus − 19.27) and average LS was lower in males (− 18.2 versus − 19.2) though differences by gender were not significant.

**Table 4:** Strain parameters in Obese adolescents and lean controls

	Obese adolescents n = 39		Lean control n = 39		P# 1	P# 2
	Mean ± SD		Mean ± SD			
Weight (kg)	99.36	27.98	59.91	12.82	< 0.0001	< 0.0001
Global long strain	− 20.98	2.83	− 23.01	2.23	0.0005	0.0045
Average longitudinal strain	− 18.87	2.55	− 20.49	2.04	0.0027	0.0140
Global circumferential strain	− 25.41	3.65	− 26.32	9.19	0.5690	0.5580
Average circumferential strain	− 22.53	3.49	− 23.84	8.50	0.3780	0.3780
Average radial strain SAX	34.29	9.80	34.41	10.07	0.9590	0.9540
Average long strain rate 4CH	− 1.02	0.21	− 1.10	0.16	0.0710	0.1080
Average CIRC strain rate SAX	− 1.33	0.20	− 1.45	0.30	0.0446	0.0701

*SAX* short axis view

P value# 1 was calculated from ANOVA test

P value# 2 was calculated from conditional logistic regression

### Strain in ONG and ODG groups

Table 5 compares 2D-STE derived global and average LS, CS and RS between the groups ONG and ODG. Global and average LS was observed to be 10% lower in the ODG group compared to the ONG group (P < 0.05). After multivariate linear regression with adjustment for confounders, namely BMI, SBP, and DBP, average LS, global CS, and average CS retained significance between both groups (P = 0.03).

**Table 5:** Strain parameters in obese adolescents with normoglycemia (ONG) and dysglycemia (ODG)

	ONG n = 25		ODG n = 14		P# 1	P# 2
	Median	IQR	Median	IQR		
Global long strain	− 21.00	3.01	− 19.18	2.75	0.0509	0.1961
Average long strain	− 18.97	3.67	− 17.87	2.49	0.0898	0.4548
Global circ strain	− 25.80	5.25	− 23.95	3.05	0.0346	0.0336
Average circ strain	− 22.47	5.80	− 20.90	1.90	0.0495	0.0324
Average radial strain	33.96	13.18	33.02	14.09	0.8005	0.7129
Average long strain rate	− 0.95	0.25	− 1.10	0.24	0.5888	0.6037
Average circ strain rate	− 1.27	0.30	− 1.32	0.25	0.7041	0.8844

*IQR* interquartile range

P value 1 was calculated from univariate linear regression; *long* = longitudinal, and *circ* = circumferential

P value # 2 was calculated from multivariate linear regression adjusted for BMI, systolic and diastolic blood pressure

Subjects in the ODG group were further partitioned into ODG with prediabetes (IFG and/or IGT) and T2DM and compared with ONG subjects. A total of 8 subjects were classified as ODG with prediabetes and 6 subjects as ODG with T2DM. Significant differences (P = 0.01) were observed in global LS between ONG subjects (− 21%), ODG with prediabetes (− 19.71%), and ODG with T2DM (− 19.1%). A similar trend was observed in average LS, in which significant differences (P = 0.04) were observed between ONG subjects (− 19%), ODG with prediabetes (− 18.6%), and ODG with T2DM (− 17.5%). In addition to significant differences in HbA1c, previously discussed, a positive correlation was observed between HbA1c and average CS (r = 0. 23; P = 0.05). Glucose and insulin for time points 0, 3, 5, 10, 30, 60, 90, 120 min during the OGTT were measured. No correlation was observed between time points for both glucose, insulin, mean glucose, mean insulin, and myocardial strain parameters, suggesting that the glycemic status was not related to myocardial deformation independent of obesity.

### Strain, strain rate, and inflammatory cytokines/lipoprotein particles

ICAM was observed to be significantly different between ONG and ODG groups (P = 0.023). Furthermore, a negative correlation was observed between average longitudinal strain rate (LSR) and anti-inflammatory adiponectin (r = − 0.41; P < 0.01) in obese adolescents.

## Discussion

Our study demonstrates that dysglycemia worsens changes in myocardial deformation parameters as noted in obese adolescents. Despite the observed changes in myocardial deformation, ventricular vascular coupling appears to be preserved in our cohort of obese, insulin resistant adolescents. Thus, clinically, our study highlights that subtle changes in myocardial function are manifest in adolescents with dysglycemia and may be useful for predicting adverse cardiovascular outcomes. “Traditional” diastolic parameters may not be sensitive to detect early stages of diastolic dysfunction. Left atrial enlargement is a reliable diastolic parameter but requires some degree of chronicity of left ventricular diastolic dysfunction to manifest. Image quality in these obese patients may limit the reliability of some of these diastolic parameters.

In our comparative cross-sectional study design, diastolic dysfunction was found to significantly higher in adolescents with dysglycemia when compared to their normoglycemic counterparts. We found significant impairments in global and average LS when compared to lean age matched controls. Furthermore, we observed that this impairment in global and average LS parameters were exaggerated in obese adolescents with dysglycemia compared to their normoglycemic counterparts. Of note in this study distinctive differences were observed in CS in dysglycemic states, implying greater abnormalities in myocardial deformation in dysglycemia than previously appreciated. This defects in CS has not been previously reported in obese insulin resistant adolescents.

Myocardial strain is well known to reflect ventricular vascular coupling, as diminished myocardial strain is known to decrease myocardial efficiency. However, in our study despite the observed changes in myocardial strain, VVI was observed to preserved. Nonetheless, VVI was additionally observed to correlate with circumferential strain rate, a load independent index of LV peak elastance and diastolic ventricular filling. Clinically, VVI is a known prognostic marker and indicator of aortic compliance, LV function, and LV performance. Together with current information regarding the prognostic value of VVI, our results suggest that ventricular efficiency is intimately dependent on diastolic ventricular function and may be a subclinical marker for changes in LV function later in life.

Prior studies have shown that in obese adolescents, insulin resistance alters myocardial substrate utilization and increases sympathetic tone, preload, and fatty acid metabolism, which ultimately results in impaired LV contractility and may lead to clinical heart failure in later life [21]. Additionally, many studies have shown that myocardial deformation as assessed by strain and strain rate are altered prior to clinically relevant ventricular dysfunction [1, 4, 22]. Singh et al. recently demonstrated that LV GLS and early diastolic strain were significantly decreased in obese adolescents and in obese subjects with nonalcoholic fatty liver disease (NAFLD) than those without NAFLD compared with appropriate controls. Furthermore, both GLS and early diastolic strain were observed to be independently associated with the homeostatic model of insulin resistance (HOMA-IR) [23]. Sanchez et al. reported that LS was found to be abnormally depressed in obese adolescents (− 13.35%) compared with lean controls (− 18.8%) [22]. Our study demonstrates similar findings in global and average LS in a population with similar demographics. As previously reported, traditional cardio metabolic risk factors, namely SBP, LDL, fasting glucose, and insulin, were not predictive of these LV functional changes in pediatric populations. Thus, this study confirmed previous reports that beyond conventional echocardiography parameters, obesity was associated with decreased myocardial strain, as assessed by 2D-STE [11, 24].

Studies of adults with T2DM have shown that cumulative effect of diabetes exposure (those with diabetes diagnosis in childhood and/or early adulthood) and BMI had greatest impact on the adverse LV remodeling [25, 26]. Bjornstad et al. were the first pediatric study to analyze strain cardiac strain from 2D-STE in adolescents with T2DM and found that compared to lean and to obese controls, adolescents with T2D had significantly lower CS [27]. Our results showcase similar findings as adolescents with dysglycemia, which included a small T2DM cohort, were observed to have a depressed CS, despite differences in study demographics, namely differences in ethnicity and a more obese cohort in our group (BMI Z = 2.49) compared to Bjornstad’s group (BMI Z = 2.01) [28, 29]. Our study thus underscores that dysglycemia attenuates unfavorable cardiac remodeling in obese adolescents, as demonstrated by changes in LS and CS. Briefly, LS is considered a measure of subendocardial injury and fibrosis, while CS is a measure of mid wall fiber damage [9]. In early ventricular dysfunction, a depressed LS is followed by an elevated CS to maintain LVEF. However, progressive dysglycemic conditions result in a decline in CS, indicating mid-wall fiber injury and damage to deeper myocardial layers. Consequently, changes in CS have been known to precede incident heart failure. In our study, we observed significant changes in both LS and CS in obese adolescents with dysglycemia compared with normoglycemic counterparts. Higher rates of dysglycemia and insulin resistance are reported in male adolescents as reflected in our study [30, 31]. Furthermore, in our cohort, no significant differences were observed in myocardial strain parameters, by gender. Bjornstad reported gender specific differences in adolescents with Type 1 diabetes (19 males and 22 females; average age 15 years). Compared to controls, adolescents with type 1 diabetes had significantly lower CS (− 20.9 vs. − 22.7%, P = 0.02), but not LS (P = 0.83). Boys with T1D had significantly lower LS than girls with T1D (− 17.5 vs. − 19.7%, P = 0.047), adjusted for Tanner stage [32]. In a study of adults (n = 277; age 56.1 years) with metabolic syndrome showed that global LS were lower in males than females as correlated with hs CRP and epicardial adipose tissue [33]. Along with the observed correlation between CS and HbA1c, we speculate that impaired glycemic control may be play a role in myocardial wall fiber injury.

Serum biomarkers of inflammation, namely adipokines and leptins, have been associated myocardial deformational change [29]. Adiponectin is an adipokine secreted by adipocytes and has been established to have a cardio protective effect, and elevated adiponectin has been associated with lower risk of myocardial infarction in men [34]. In adiponectin knockout mouse models, abnormal cardiac remodeling and cardiac concentric hypertrophy have been observed, indicating adiponectin mediated signaling may play a role in preventing abnormal ventricular remodeling [35, 36]. In our cohort we demonstrated a negative linear relationship between average LS rate and adiponectin (r = − 0.4), indicating the serum adiponectin levels correlate with early subendocardial fiber damage.

Previously, Li et al. reported that LS at the cardiac apex correlated with VVI in adults with T2DM with either normal or depressed ejection fraction. Our study demonstrated similar findings. However, Li et al. demonstrated abnormal VVI (1.55 ± 0.53), indicating impaired ventricular-vascular coupling, a finding not observed in our cohort. We speculate that changes in VVI in pediatric populations likely are less pronounced compared to adult populations due to lack of other known comorbidities that abnormally alter ventricular-vascular coupling.

Of note is the correlation observed in our study between VVI and circumferential strain rate, a proxy for LV peak elastance. To our knowledge, this is the first study to have correlated VVI, an index of mechanical efficiency and myocardial deformation, namely circumferential strain rate. In the broader context of myocardial mechanics and function, strain rate is a load independent marker of myocardial deformation and can be utilized for clinical assessment of regional wall motion abnormalities [37–39]. While strain rate is unable to quantify myocardial contractility due to its load independence, it remains a clinically useful tool for assessment of passive expansion and recoil of the myocardium [39]. Clinically, measurements of strain rate have correlated well with improvements in wall motion deformation post coronary bypass and percutaneous revascularization and for identification of wall motion abnormalities in hypertrophic cardiomyopathy and cardiac amyloidosis [39–41]. In T2DM, myocardial steatosis and myocardial triglyceride content have demonstrated to be independent predictors of both LV and RV longitudinal strain and strain rate, suggesting that subclinical changes in myocardial deformation assessed by strain rate are influenced by extra myocardial signaling factors [42]. In the metabolic phenotype of heart failure with preserved ejection fraction (HFpEF), similar reductions in diastolic strain rate have been observed [43, 44]. Together with the observed depression in CSR in dysglycemia in study, these data suggest broader implications for the importance of myocardial strain rate in understanding the relationship between dysglycemia and abnormalities in myocardial deformation, particularly in HFpEF in patients with T2DM or metabolic syndrome,

### Limitations

Our study has several limitations. Due to the cross-sectional design, causal relationships cannot be established among the observed associations. Additionally, the predominance of Hispanic subjects, about 60% of the cohort, limits generalizability of the findings. However, the study presented simultaneously provides novel data in for pediatric Hispanic subjects, a population known to be a high risk for diabetes. The reduced sample size in this study reduced power required to detect differences between groups based on OGTT results. Though we had age and gender matched control group, we recognize that the control group was predominantly Caucasian.

## Conclusion

Obese adolescents especially those with dysglycemia demonstrated greater abnormalities in myocardial deformation than previously appreciated in the literature. Furthermore, significant differences in strain in the context of dysglycemia and observed correlations between strain rate and HbA1c suggest that strain rate may be a reliable marker to predict adverse cardiac remodeling in overt diabetes. In conclusion, our study highlights that subtle functional changes are manifest in adolescents with dysglycemia and may be useful for predicting adverse cardiovascular outcomes much earlier compared to their lean contemporary peers.

## Data Availability

All data generated or analysed during this study are included in this published article.

## Abbreviations

LS: longitudinal strain
CS: circumferential strain
VVI: ventricular vascular coupling index
ESP: end systolic pressure (0.9× systolic blood pressure)
Ees: end systolic elastance
Ea: arterial elasticity
MVE/A: E/A wave ratio, a diastolic index
LVM (5/6AL): LV mass indexed to BSA using 5/6 area length measurement
LVMI (m mode): LV mass indexed to BSA
